# Correction: CAPS-1 promotes fusion competence of stationary dense-core vesicles in presynaptic terminals of mammalian neurons

**DOI:** 10.7554/eLife.12968

**Published:** 2015-11-18

**Authors:** Margherita Farina, Rhea van de Bospoort, Enqi He, Claudia M Persoon, Jan RT van Weering, Jurjen H Broeke, Matthijs Verhage, Ruud F Toonen

Margherita F, van de Bospoort R, Enqi H, Persoon CM, van Weering JRT, Broeke JH, Verhage M, Toonen RF. 2015. CAPS-1 promotes fusion competence of stationary dense-core vesicles in presynaptic terminals of mammalian neurons. *eLife* **4**:e05438. doi: 10.7554/eLife.05438

Published 26 February 2015

An error was identified in Figure 6. Figure 6E originally showed the control mEPSC typical trace twice: once for the control and, incorrectly, once for the CAPS-EYFP rescue. The error arose during the final stages of manuscript preparation, when selecting typical traces; one control trace was inadvertently transferred twice from our analysis software to the final Adobe Illustrator file and mislabeled manually in the Illustrator file. The duplicate traces were then selected for the control and incorrectly also for the rescue. We have corrected this and now show the correct typical mEPSC trace for the CAPS-EYFP rescue in Figure 6E.

The corrected Figure 6 is shown here:

**Figure 1:**
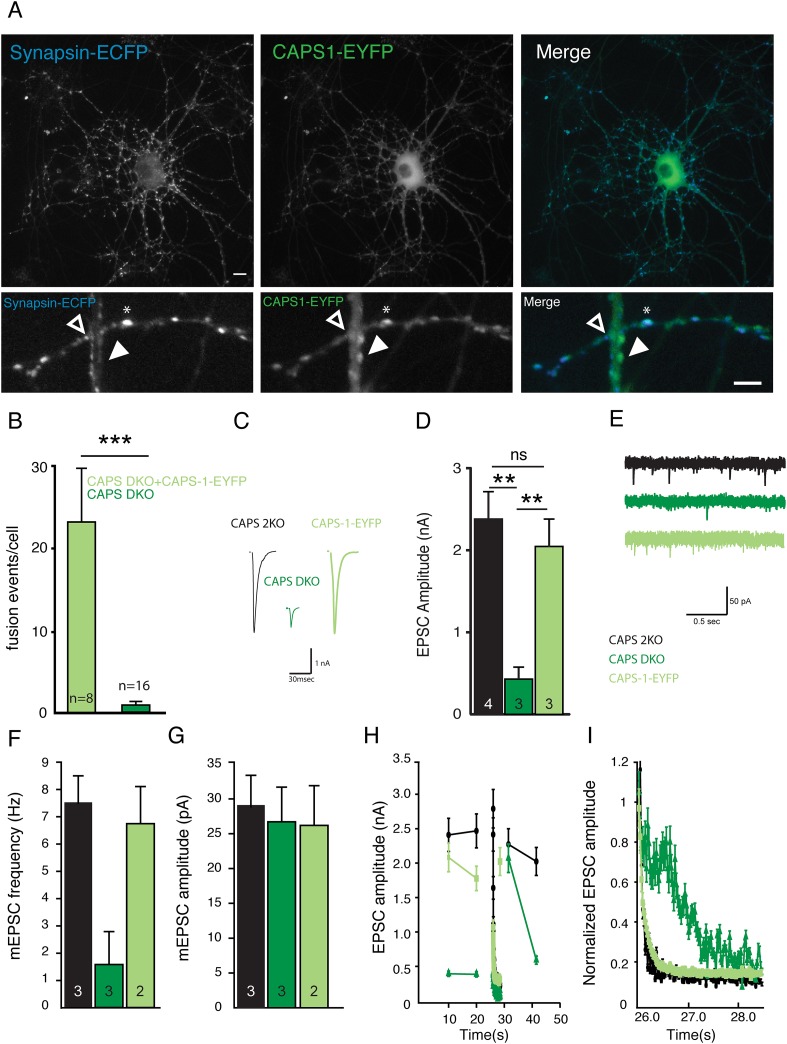

The originally published Figure 6 is also shown for reference:

**Figure 2:**
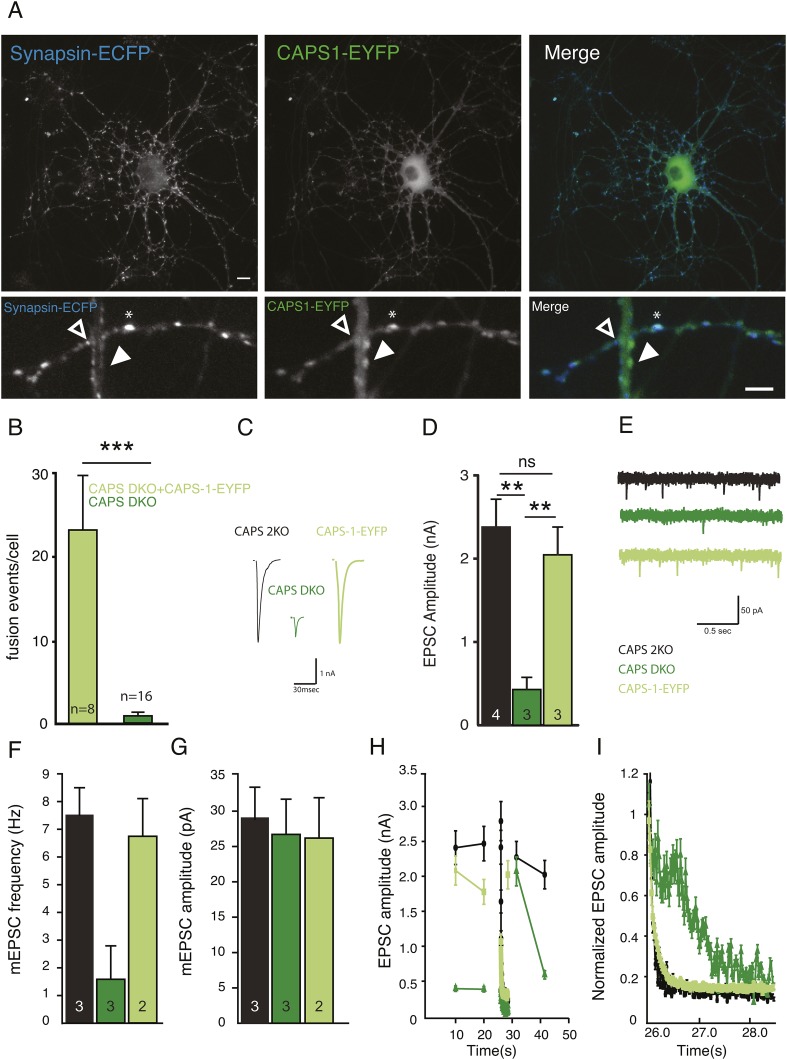

The article has now been corrected.

